# Corneal *in vivo* Confocal Microscopy for Assessment of Non-Neurological Autoimmune Diseases: A Meta-Analysis

**DOI:** 10.3389/fmed.2022.809164

**Published:** 2022-03-09

**Authors:** Yuxiang Gu, Xin Liu, Xiaoning Yu, Qiyu Qin, Naiji Yu, Weishaer Ke, Kaijun Wang, Min Chen

**Affiliations:** ^1^Eye Center of the Second Affiliated Hospital, School of Medicine, Zhejiang University, Hangzhou, China; ^2^Zhejiang Provincial Key Lab of Ophthalmology, Hangzhou, China

**Keywords:** corneal nerve, confocal microscopy, non-neurological autoimmune diseases, type 1 diabetes, Sjögren's syndrome

## Abstract

**Purpose:**

This study aimed to evaluate the features of corneal nerve with *in vivo* confocal microscopy (IVCM) among patients with non-neurological autoimmune (NNAI) diseases.

**Methods:**

We systematically searched PubMed, Web of Science, and Cochrane Central Register of Controlled Trials for studies published until May 2021. The weighted mean differences (WMDs) of corneal nerve fiber length (CNFL), corneal nerve fiber density (CNFD), corneal nerve branch density (CNBD), tortuosity, reflectivity, and beadings per 100 μm with a 95% CI between NNAI and control group were analyzed using a random-effects model.

**Results:**

The results showed 37 studies involving collective totals of 1,423 patients and 1,059 healthy controls were ultimately included in this meta-analysis. The pooled results manifested significantly decreased CNFL (WMD: −3.94, 95% CI: −4.77–−3.12), CNFD (WMD: −6.62, 95% CI: −8.4–−4.85), and CNBD (WMD: −9.89, 95% CI: −14–−5.79) in NNAI patients. In addition, the NNAI group showed more tortuous corneal nerve (WMD: 1.19, 95% CI:0.57–1.81). The comparison between NNAI patients and healthy controls in beadings per 100 μm corneal nerve length was inconsistent. No significant difference was found in the corneal nerve fiber reflectivity between NNAI and the control group (WMD: −0.21, 95% CI: −0.65–0.24, *P* = 0.361).

**Conclusions:**

The parameters and morphology of corneal nerves observed by IVCM proved to be different in NNAI patients from healthy controls, suggesting that IVCM may be a non-invasive technique for identification and surveillance of NNAI diseases.

## Introduction

Autoimmune diseases are a range of diseases characterized by increased activity of the immune system which results in organ damage or dysfunction (1). According to research, autoimmune diseases affect approximately 7.6–9.4% of the general population and impose huge burdens not only on patients themselves but also on the whole society (2). Genetic, microbial, environmental, lifestyle, and psychological factors are thought as contributing elements to autoimmune diseases although the underlying etiology remains to be explored (3). Despite impressive advances in the management of autoimmune diseases, they are still impossible to cure. A definitive diagnosis as early as possible can increase the efficiency and efficacy of the treatment strategy and also help to avoid complications (4, 5). In this case, an early diagnosis can play a decisive role in improving the patient's quality of life as well as life expectancies.

The cornea is a transparent part covering the front portion of the eyewall and is regarded as the most densely innervated tissue in the human body. With a density of approximately 7,000 epithelial-free nerve endings per square millimeter, the cornea is about 300–600 times more sensitive than skin (6). A review has concluded that changes in corneal innervation can occur for many reasons, including keratitis, corneal dystrophies, corneal degenerations, corneal ecstasies, glaucoma, medical treatment, etc (7). Corneal nerve alternation is not only a window to observe some ocular diseases, but also a potential window to observe systemic diseases. In this article, we focus on non-neurological autoimmune (NNAI) diseases which exclude autoimmune diseases that affect the central nervous system mostly or present obvious psychiatric manifestations. This is a range of autoimmune diseases admitted by the American Autoimmune and Related Diseases Association and excluded from the list of known neurological disorders by the American Academy of Neurology. Some of the NNAI diseases have been discovered to be associated with the human cornea and peripheral neurological manifestations as early as the 1980's. Keratitis was found may be a presenting sign of rheumatoid arthritis or sarcoidosis (8); immune deposits in the cornea were found in patients with systemic lupus erythematosus by immunopathological staining (9). People with NNAI diseases are at high risk of innervation alternation and have a high incidence of various kinds of neuropathy. For instance, it is reported that up to 86% of patients with sarcoidosis present with typical small-fiber neuropathy symptoms (10), over 60% of patients with Sjögren's syndrome suffer from peripheral neuropathy (11, 12), higher prevalence of NNAI diseases including rheumatoid arthritis, systemic lupus erythematosus, Sjögren's syndrome suffer from fibromyalgia and so on (13). Innervation alternation may be progressing soon after the onset of NNAI because of the high sensitivity of the nerves. The corneal nerve may have undergone a long time when observable changes appear, but no symptoms or discomfort are perceived by the patient. For this reason, corneal signs may be the first manifestation of autoimmune diseases. Alteration of corneal nerve parameters is of great significance beyond ocular diseases, it can provide clinicians with thought-provoking insight into the clinical diagnosis or management of many diseases like type 1 diabetes, Parkinson's disease, Friedreich ataxia before organ damages is manifested (14–16). Many researchers showed significant associations between the reduction in corneal innervation and increasing disease severity in neurological autoimmune diseases like multiple sclerosis (17, 18). However, studies present conflicting results on the effect of NNAI diseases on corneal innervation. Moreover, previous studies focus on histopathology results rather than non-invasive analysis. *In vivo* confocal microscopy (IVCM), with its ease of clinical set-up and a 800-fold magnification of cellular level, is becoming a promising as well as a non-invasive tool to view and quantify corneal nerve parameters directly (19). In this way, IVCM may provide a non-invasive potential biomarker for NNAI. Hence, we collected data from different studies about the corneal nerve parameters measured by IVCM in various NNAI diseases and conduct a meta-analysis to evaluate the potential application of this technique as an indicator of NNAI diseases.

## Methods

### Search Strategy

**Figure 1:**
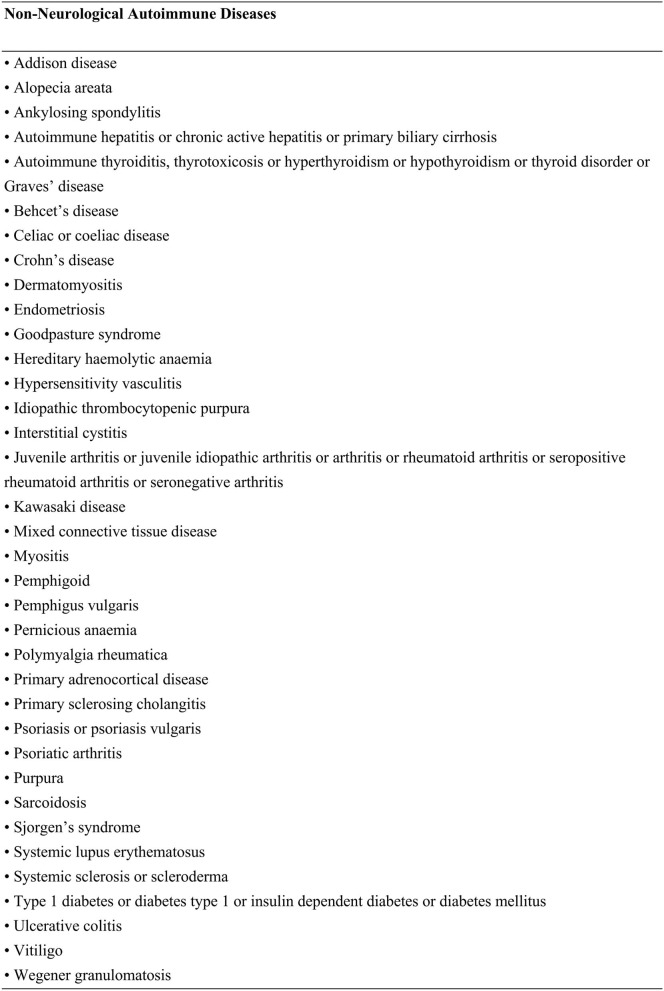
Search terms used to identify non-neurological autoimmune diseases.

A systematic literature search was conducted in PubMed, Web of Science, and Cochrane Central Register of Controlled Trials (updated to May 2021). No constraints were applied regarding the language or the publication time of works of literature. Search terms included confocal microscopy or IVCM or cornea^*^ nerve with a combination of autoimmune diseases or autoimmune diseases or XXX, the last-mentioned representing 36 individual NNAI diseases (Figure 1). The selection of NNAI diseases referred to Alexis E. Cullen's study (20). All autoimmune diseases searched were selected a priori from the American Autoimmune and Related Diseases Association and were cross-checked against known neurological disorders, as listed by the American Academy of Neurology. We excluded uveitis for it is essentially a type of eye disease and would, to some extent, affects corneal structure and function. Neither did we adopt data among type 1 diabetes peripheral neuropathy and type 1 diabetic retinopathy, for they had been proved to be related to corneal nerve changes (21–23).

### Inclusion and Exclusion Criteria

We included studies that met the following criteria: (1) at least 10 adults with a definite diagnosis of NNAI diseases in the test group; (2) a healthy population as the control group; (3) reporting at least corneal nerve fiber density (CNFD) or corneal nerve fiber length (CNFL). Exclusion Criteria were as follows: (1) inappropriate types of articles, such as reviews, case reports, editorials, conference papers and abstracts, short surveys, or letters; (2) studies which subjects with NNAI diseases were divided into irrelevant subgroups, for instance, dividing patients with type 1 diabetes by erectile dysfunction; (3) studies assessing only animals; (4) studied based or partially based on the same population (studies with the most sufficient data were selected); (5) articles without sufficient data (i.e., mean and *SD*).

### Data Extraction

All publications searched were exported to Endnote (version X9.3; The Thomson Corporation Corp, Stanford, CT, USA). Then, duplicate publications were collated and removed. Two researchers (YG and XL) assessed the titles and abstracts independently for potential eligibility, and the full-text articles were retrieved which appeared relevant. Final eligibility was performed by assessing full-text articles and disagreements on eligibility were resolved via discussion and, if necessary, by consulting a third researcher (XY). Studies that were in accord with the inclusion/exclusion criteria were read, and the following information was extracted from the eligible articles: study details (such as the first author's name, year of acceptance, type of IVCM, and software used to measure corneal nerve parameters) and subjects' information (such as mean age, subjects' sex, duration of NNAI diseases, type of diseases, and corneal nerve parameters). The screening process and protocol are summarized and described in the flow diagram.

### Assessments of Article Quality

The Newcastle-Ottawa Scale, covering three methodological domains (selection criteria, comparability, and measurement of exposure and/or outcome), was used to rate article quality. With a maximum score of 9, we defined the article as low quality if the numeric score was 0–3, moderate quality if the score was 4–6, and high quality if the score was 7–9. Low-quality articles were excluded.

### Statistical Analysis

This meta-analysis was conducted using the Stata (version 15.1; StataCorp LLC, College Station, TX, USA), a *p*-value of <0.05 was considered statistically significant. We extracted the mean, standard deviation, and sample size for continuous corneal nerve parameters, and the Random-effects model was applied to calculate the weighted mean difference (WMD) with 95% CI. In order to facilitate comparison, we defined the total length of the corneal nerve fibers as CNFL, the total number of corneal nerve fibers per mm^2^ as CNFD, and the number of branches originating from major nerve trunks per mm^2^ as corneal nerve branch density (CNBD). Nerve length or nerve density was divided by image area, if necessary, in order to unify the units of corneal nerve parameters. Besides the parameters above, we also recorded nerve tortuosity, reflectivity, and beadings. Nerve tortuosity and nerve reflectivity were presented as four grades according to previously validated grading scales (24). Beadings were defined as the number of bead-like formations in 100 μm of the nerve fiber. It should be mentioned that some works of research evaluated corneal nerve tortuosity with tortuosity coefficient, which is not adopted in this meta-analysis for a reliable comparison. We performed a sensitivity analysis by omitting one study at a time and calculating a pooled estimate for the remaining studies to evaluate the contribution of each individual study to the results. The I^2^ statistic was used to estimate heterogeneity among the studies. To explore the potential confounding factors, we performed subgroup analysis by age, type of IVCM, software used to measure corneal nerve parameters, and types of NNAI diseases. Publication bias was estimated by funnel plot, as well as Egger's linear regression test and Begg's rank association test with significance set to *P* < 0.1 (25, 26).

## Results

### Search Process

**Figure 2:**
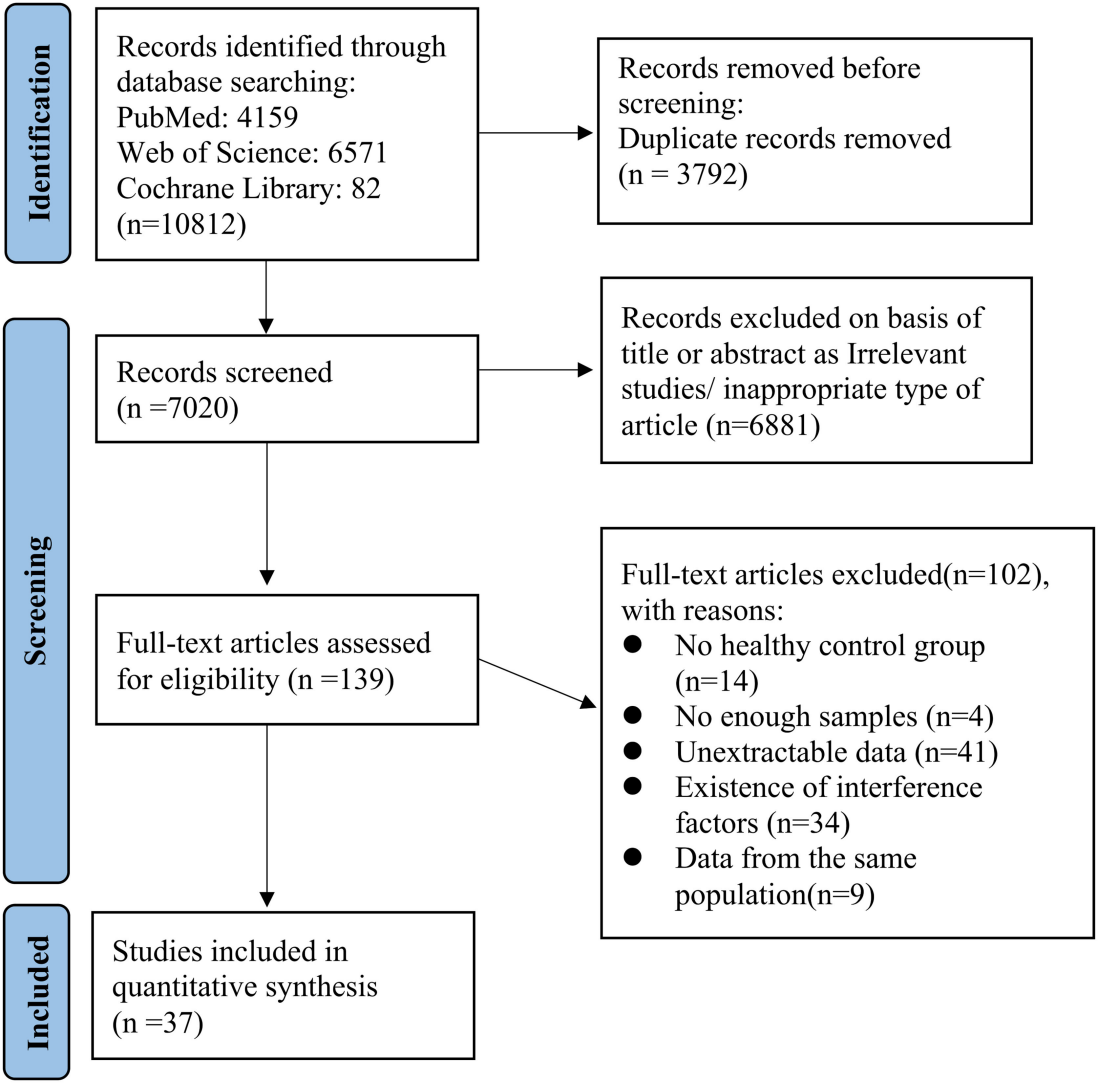
Flow diagram of article selection.

The selection of studies is shown in Figure 2. Potential references were screened from PubMed (*n* = 4,159), Web of Science (*n* = 6,571) and Cochrane Library (*n* = 82). After duplicate publications were removed, the titles and abstracts of 7,020 remaining studies were assessed for potential eligibility. For final eligibility, a total of 139 full-text articles were screened thoroughly and 102 studies were excluded due to reasons listed in Figure 2. No articles were excluded because of low quality. Quality rating scores ranged from 5 to 9 (mean: 7.24, *SD*: 1.04). Thus, a total of 37 studies were eligible for the final meta-analysis which included 1,423 patients and 1,059 healthy controls (16, 27–61).

### Study Characteristics

**Figure 3:**
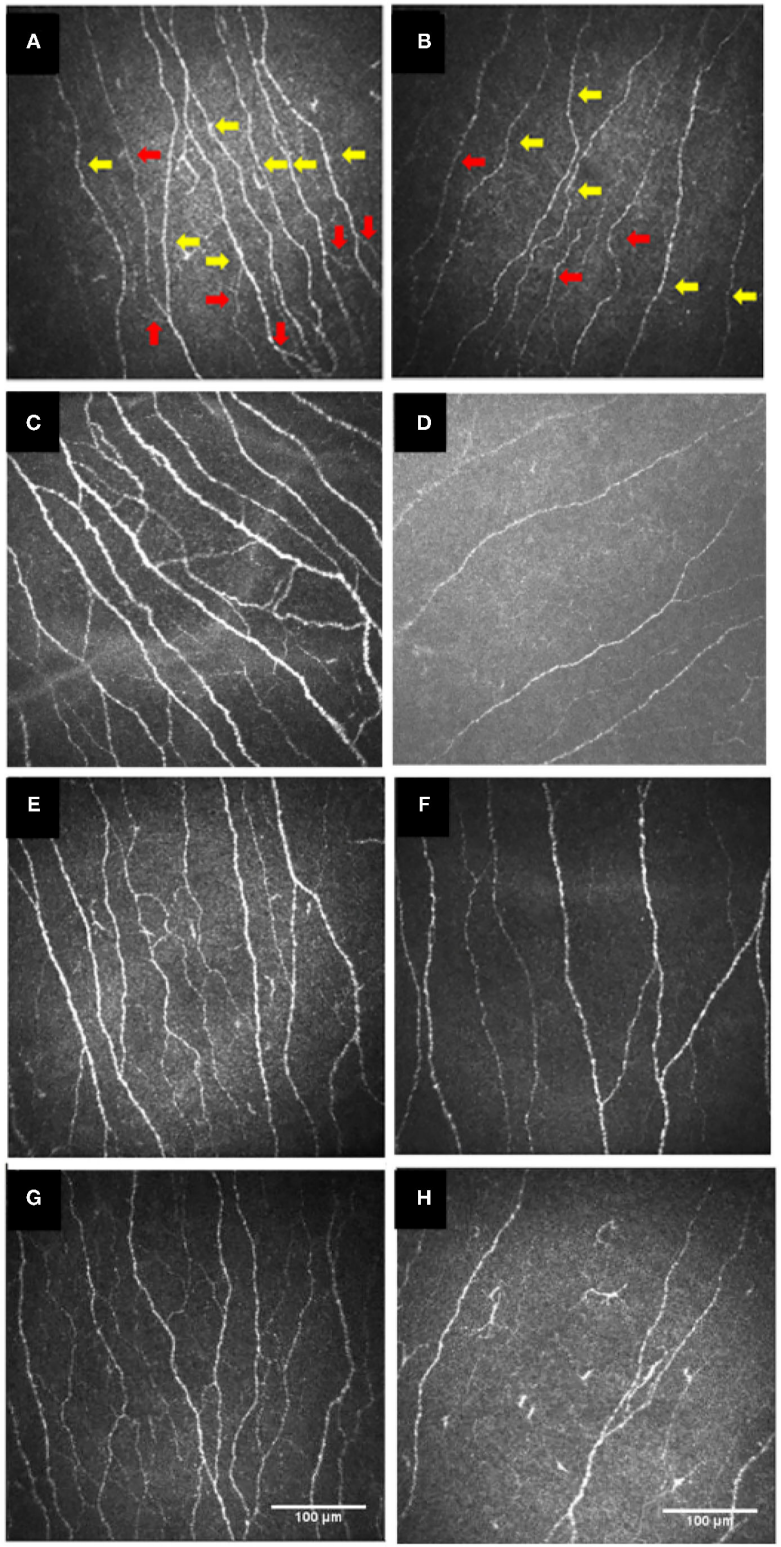
IVCM images of the cornea in the healthy controls **(A,C,E,G)** and patients with type 1 diabetes **(B)**, or with Sjögren's syndrome **(D)**, or with celiac disease **(F)**, or with Behçet's disease **(H)**. Red arrows show corneal nerve branches and yellow arrows show corneal nerve fibers. **(A,B)** were re-organized with permission from (45), copyright 2017, Public Library of Science. **(C,D)** were re-organized with permission from (62), copyright 2021, BioMed Central. **(E,F)** were re-organized with permission from (33), copyright 2020, Public Library of Science. **(G,H)** were re-organized with permission from (40), copyright 2018, Frontiers. IVCM, *in vivo* confocal microscopy.

Among the 37 included studies, 18 were related to type 1 diabetes, 13 were related to Sjögren's Syndrome, 1 was related to Bechet's disease, 1 was related to coeliac disease, 1 was related to Graves' disease, 1 was related to hypothyroidism, 1 was related to mucous membrane pemphigoid, and 1 was related to rheumatoid arthritis. As shown in Table 1, different studies reported different corneal nerve parameters. Most of the studies used laser scanning confocal microscopy (LSCM) or slit scanning confocal microscopy (SSCM) as IVCM appliances, except for tandem scanning confocal microscopy (TSCM) in one study and unspecified appliance in another. As for IVCM image analysis software, CCMetrics, ACCMetrics, and Image J were commonly used. Other characteristics of the included studies such as demographics, research groups, disease durations are also summarized in Table 1. Representative IVCM images of the cornea in patients with healthy controls and patients with NNAI diseases are listed in Figure 3.

**Table 1:** Characteristics of the included studies (*n* = 37).

**References**	**Country**	**Duration (Years)**	**Groups**	**Number**	**Age**	**Type of IVCM**	**Sex (F/M)**	**Software used**	**Quality scores**	**CN**			**T**	**B**	**R**
										**FD**	**FL**	**BD**			
Ahmed et al. (54)	Canada	17.60 ± 14.00	Type 1 diabetes	56	34.90 ± 14.80	LSCM	29/27	CCMetrics	7	√	√	√	√		
		–	Healthy controls	64	38.90 ± 17.60		34/30								
Alam et al. (45)	UK	17.20 ± 12.00	Type 1 diabetes	30	38.80 ± 12.50	LSCM	17/13	CCMetrics	6	√	√	√			
		–	Healthy controls	27	41.00 ± 14.90		11/16								
Ceská Burdová et al. (39)	Czech Republic	13.50 ± 7.20	Type 1 diabetes	24	37.70 ± 12.30	SSCM	NA	Built-in software	7	√	√	√	√		
		–	Healthy controls	20	32.20 ± 9.90		11/9								
Chen et al. (38)	UK	20.00 ± 11.10 11.10	Type 1 diabetes	63	44.00 ± 15.00	LSCM	NA	CCMetrics	5	√	√	√			
		–	Healthy controls	84	46.00 ± 15.00		NA								
Chen et al. (50)	UK	23.00 ± 15.00	Type 1 diabetes	46	44.00 ± 13.00	LSCM	NA	CCMetrics	9	√	√	√			
		–	Healthy controls	26	44.00 ± 15.00		NA								
Cozzini et al. (29)	Italy	8.70 ± 4.20	Type 1 diabetes	150	16.60 ± 4.00	LSCM	77/73	ACCMetrics	7	√	√	√			
		–	Healthy controls	51	16.30 ± 2.90		25/26								
D'Onofrio et al. (28)	Italy	19.40 ± 7.60	Type 1 diabetes	25	53.30 ± 11.70	LSCM	8/17	CCMetrics	6		√				
		–	Healthy controls	23	54.10 ± 11.10		12/11								
Ferdousi et al. (35)	UK	9.10 ± 2.70	Type 1 diabetes	64	14.60 ± 2.50	LSCM	31/33	CCMetrics	8	√	√	√	√		
		–	Healthy controls	55	13.60 ± 3.10		33/22								
Ferdousi et al. (37)	USA	29.98 ± 2.64	Type 1 diabetes	42	49.21 ± 2.53	LSCM	15/27	ACCMetrics	6	√	√	√			
		–	Healthy controls	25	48.70 ± 2.84		14/11								
Gad et al. (27)	Qatar	4.08 ± 2.91	Type 1 diabetes	20	14.47 ± 2.43	LSCM	NA	CCMetrics	8	√	√	√	√		
		–	Healthy controls	20	12.83 ± 1.91		NA								
Hertz et al. (55)	Canada	NA	Type 1 diabetes	12	NA	LSCM	NA	CCMetrics	8	√	√	√	√		
		–	Healthy controls	20	41.40 ± 17.30		15/5								
Schiano Lomoriello et al. (34)	Italy	12.47 ± 8.29	Type 1 diabetes	19	37.42 ± 8.99	SSCM	10/9	CS4 software	8	√	√	√	√	√	
		–	Healthy controls	19	40.31 ± 11.15		10/9								
Misra et al. (48)	New Zealand	25.8 ± 11.3	Type 1 diabetes	53	48.60 ± 11.80	LSCM	27/26	Analysis 3.1	7		√				
		–	Healthy controls	40	44.30 ± 14.70		23/17								
Pritchard et al. (52)	Australia	20.00 ± 15.00	Type 1 diabetes	168	43.00 ± 16.00	LSCM	83/85	CCMetrics	8		√	√			
		–	Healthy controls	154	46.00 ± 15.00		84/70								
Scarr et al. (43)	Canada	23.50 ± 14.40	Type 1 diabetes	139	42.00 ± 16.00	LSCM	73/66	CCMetrics	7		√				
		–	Healthy controls	68	38.00 ± 16.00		36/32								
Stem et al. (51)	USA	13.50 ± 6.70	Type 1 diabetes	25	38.70 ± 14.20	LSCM	18/7	Image J	8		√				
		–	Healthy controls	9	43.90 ± 10.20		6/3								
Szalai et al. (16)	Hungary	5.79 ± 2.58	Type 1 diabetes	18	16.45 ± 2.59	LSCM	NA	ACCMetrics	5	√	√	√			
		–	Healthy controls	17	26.53 ± 2.43		8/9								
Tummanapalli et al. (31)	Australia	15.00 ± 9.00	Type 1 diabetes	27	32.00 ± 10.00	LSCM	10/17	ACCMetrics	8	√	√	√			
		–	Healthy controls	29	37.00 ± 11.00		13/16								
Barcelos et al. (30)	Portugal	11.70 ± 7.70	Sjögren's Syndrome	55	57.80 ± 11.80	LSCM	NA	Image J	8	√	√				
		–	Healthy controls	20	51.00 ± 6.50		NA								
Castillo et al. (59)	Spain	8.60 ± 3.20	Sjögren's Syndrome	11	61.30 ± 11.30	SSCM	10/1	NA	8		√	√		√	
		–	Healthy controls	10	65.40 ± 3.20		8/2								
Benítez del Castillo et al. (60)	Spain	10.40 ± 3.20	Sjögren's Syndrome	11	52.90 ± 8.70	SSCM	10/1	NA	8		√	√	√	√	√
		–	Healthy controls	10	68.70 ± 7.10		8/2								
Chen et al. (56)	China	NA	Sjögren's Syndrome	26	42.30 ± 9.70	LSCM	25/1	Analysis 3.1	7	√			√		
		–	Healthy controls	26	40.80 ± 9.30		21/5								
Levy et al. (44)	France	NA	Sjögren's Syndrome	30	58.90 ± 15.40	LSCM	20/10	Image J	6	√	√		√		√
		–	Healthy controls	15	59.30 ± 12.30		9/6								
Matsumoto et al. (32)	Japan	NA	Sjögren's Syndrome	23	65.40 ± 11.40	LSCM	23/0	Image J	8	√	√		√	√	√
		–	Healthy controls	13	68.80 ± 9.80		13/0								
McNamara et al. (47)	USA	NA	Sjögren's Syndrome	10	56.50 ± 8.71	SSCM	9/1	CC Metrics	8	√	√	√	√		
		–	Healthy controls	10	58.20 ± 8.44		9/1								
Semeraro et al. (46)	Italy	12.29 ± 6.37	Sjögren's Syndrome	24	54.31 ± 11.49	NA	24/0	Image J	7	√	√	√	√	√	
		–	Healthy controls	24	48.88 ± 6.50		24/0								
Tepelus et al. (42)	USA	NA	Sjögren's Syndrome	22	57.50 ± 8.60	LSCM	21/1	Image J	8		√		√		√
		–	Healthy controls	7	59.30 ± 12.70		6/1								
Tuisku et al. (57)	Finland	NA	Sjögren's Syndrome	20	54.50 ± 7.00	SSCM	19/1	Built-in software	5	√					
		–	Healthy controls	10	49.80 ± 5.00		9/1								
Tuominen et al. (61)	Finland	8.00 ± 4.60	Sjögren's Syndrome	10	50.10 ± 13.50	TSCM	9/1	NA	6	√					
		–	Healthy controls	10	48.30 ± 14.50		9/1								
Villani et al. (53)	Italy	NA	Sjögren's Syndrome	15	52.10 ± 15.40	LSCM	11/4	Cell Count software	8	√			√	√	
		–	Healthy controls	15	45.20 ± 15.90		10/5								
Villani et al. (58)	Italy	NA	Sjögren's Syndrome	15	52.30 ± 10.30	SSCM	12/3	Cell Count software	8	√			√		√
		–	Healthy controls	20	51.20 ± 18.20		13/7								
Bitirgen et al. (40)	Turkey	NA	Bechet's disease	49	39.90 ± 11.20	LSCM	32/17	ACCMetrics	8	√	√	√			
		–	Healthy controls	30	41.20 ± 11.50		20/10								
Gad et al. (33)	Qatar	4.49 ± 4.02	Coeliac disease	20	11.78 ± 1.74	LSCM	NA	CCMetrics	8	√	√	√	√		
		–	Healthy controls	20	12.83 ± 1.91		NA								
Kocabeyoglu et al. (49)	Turkey	0.87 ± 0.63	Graves' disease	40	35.40 ± 11.20	SSCM	29/11	Image J	7	√	√	√	√		
		–	Healthy controls	40	33.80 ± 10.30		26/14								
Sharma et al. (36)	UK	NA	Hypothyroidism	20	49.55 ± 13.34	LSCM	11/9	CCMetrics	8	√	√	√			
		–	Healthy controls	20	44.95 ± 14.29		12/8								
Tepelus et al. (41)	USA	NA	Mucous Membrane Pemphigoid	23	76.40 ± 13.80	LSCM	NA	Image J	8		√		√		√
		–	Healthy controls	8	74.30 ± 7.50		NA								
Barcelos et al. (30)	Portugal	11.70 ± 7.70	Rheumatoid arthritis	18	55.30 ± 13.70	LSCM	NA	Image J	6	√	√				
		–	Healthy controls	20	51.00 ± 6.50		NA								

*F/M, female/male; CN, corneal nerve; FD, fiber density; FL, fiber length; BD, branch density; T, tortuosity; B, beadings; R, reflectivity; NA, not available*.

### Corneal Nerve Parameters (CNFL, CNFD, CNBD)

**Figure 4:**
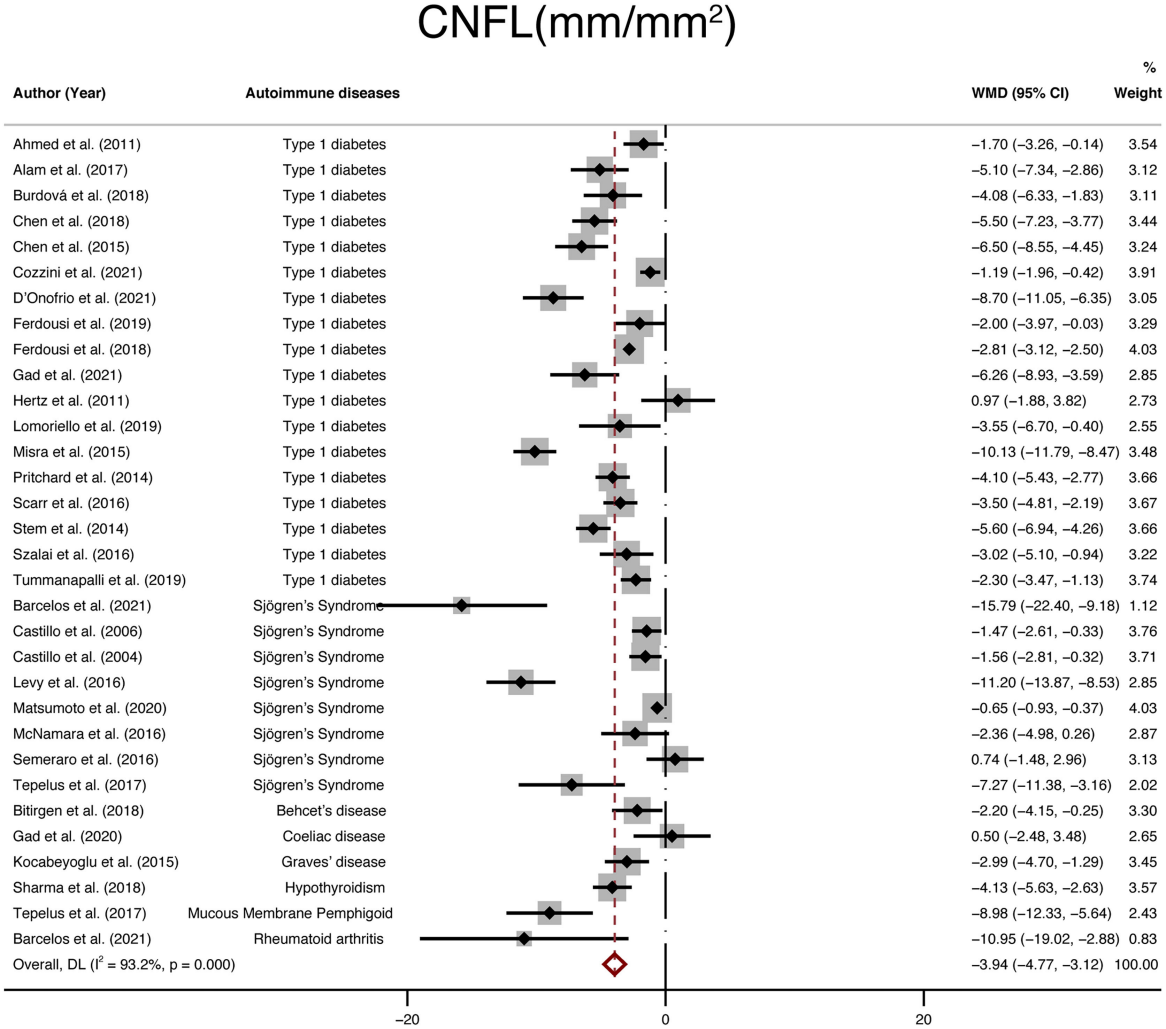
Forest plot of the WMD of CNFL between the NNAI group and the control group. WMD, weighted mean difference; CI, confidence interval; CNFL, corneal nerve fiber length; NNAI, non-neurological autoimmune (diseases).

Including 2,335 participants (1,337 in the NNAI group and 998 in the control group), thirty-two studies reported on CNFL. The WMD in CNFL between NNAI and control groups was−3.94 (95% CI: −4.77–−3.12, *P* < 0), with significant heterogeneity across studies (I^2^ = 93.2%, Figure 4). The results showed CNFL (mm/mm^2^) was obviously lower in the NNAI group.

**Figure 5:**
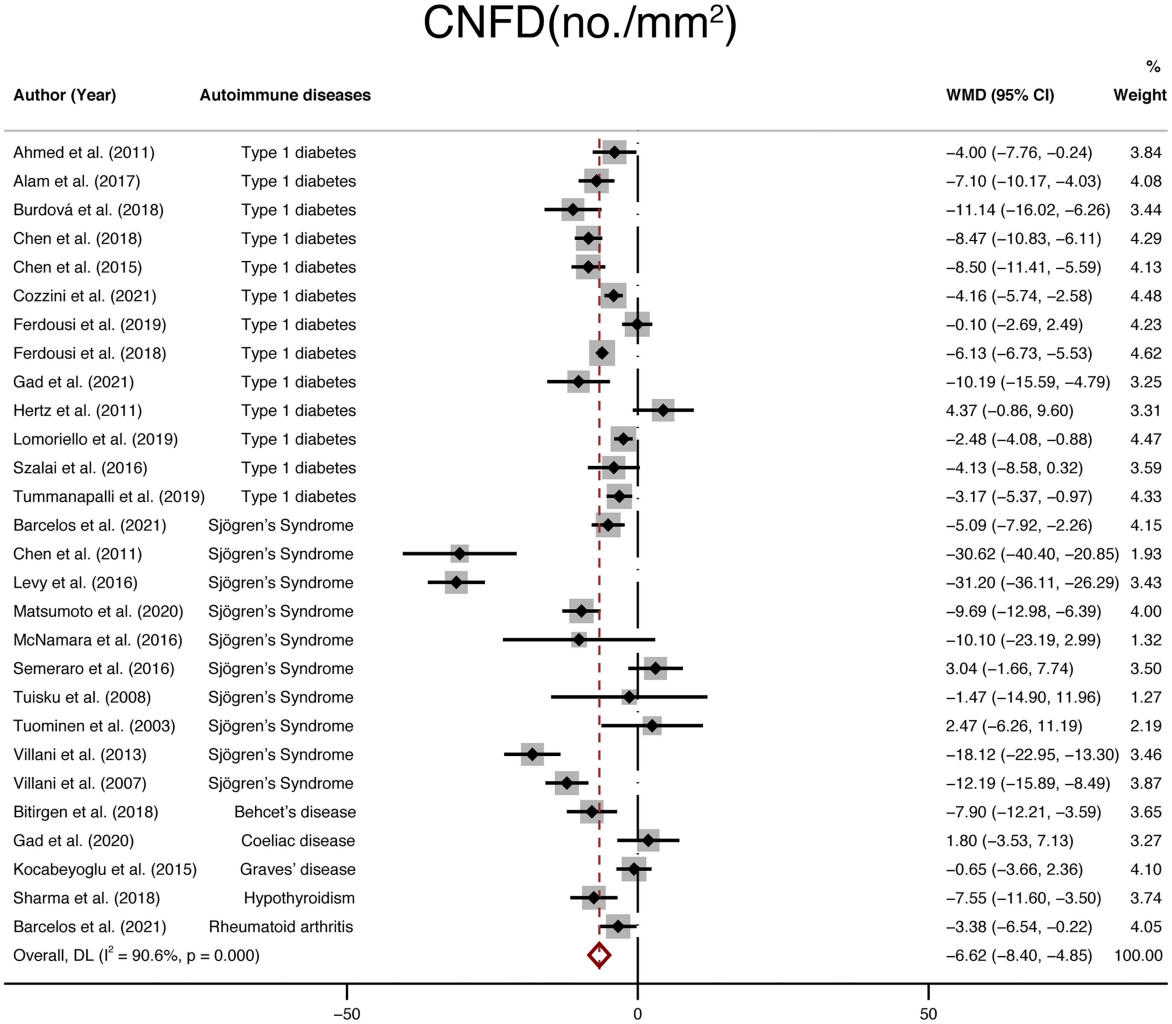
Forest plot of the WMD of CNFD between the NNAI group and the control group. WMD, weighted mean difference; CI, confidence interval; CNFD, corneal nerve fiber density; NNAI, non-neurological autoimmune (diseases).

Furthermore, twenty-eight studies with a total of 1,696 participants (946 in the NNAI group and 750 in the control group) reported on CNFD. The weighted mean difference was−6.62 (95% CI: −8.4–−4.85, *P* < 0), with significant heterogeneity across studies (I^2^ = 90.6%, Figure 5), showing that CNFD (no./mm^2^) of the NNAI group was significantly lower than that of the control group.

**Figure 6:**
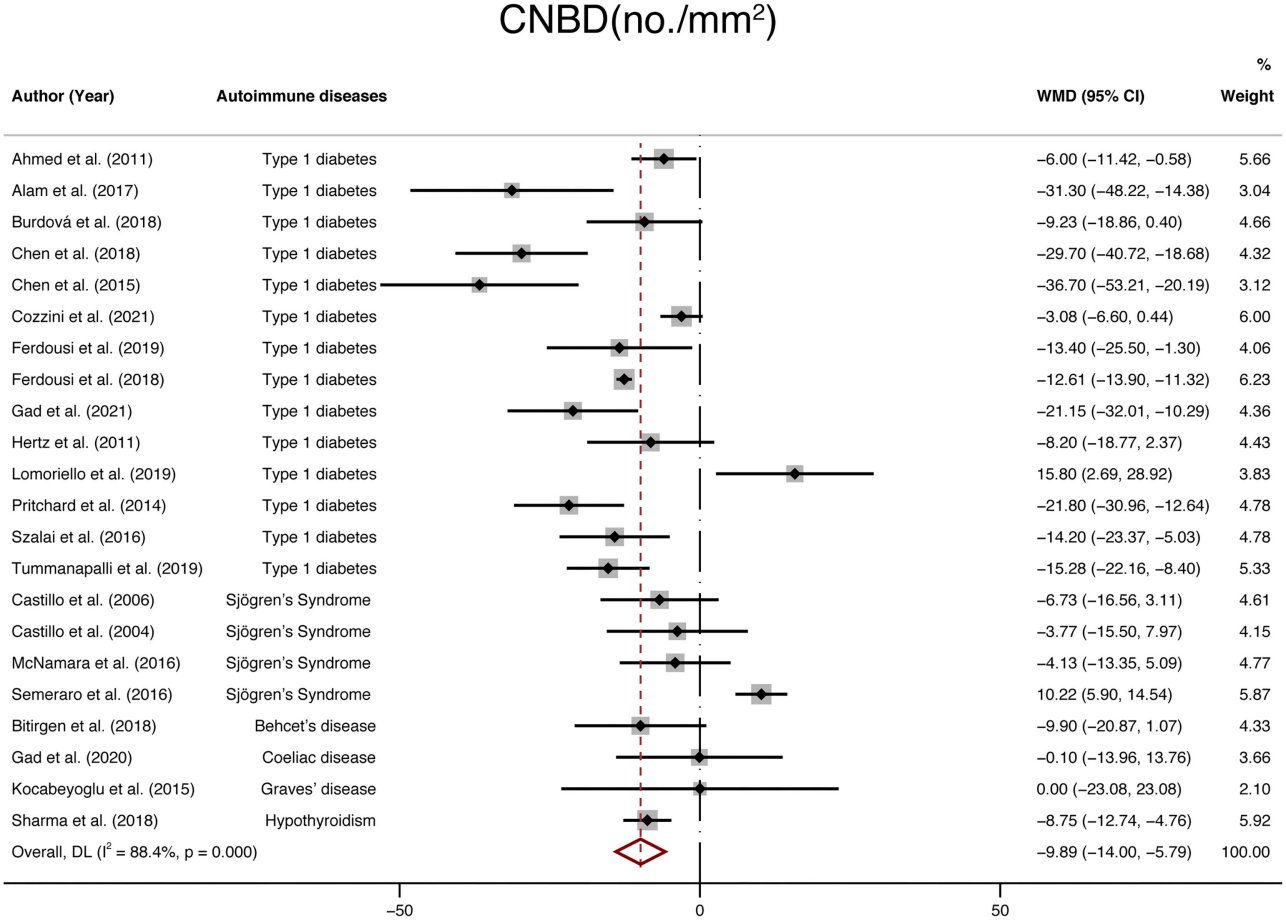
Forest plot of the WMD of CNBD between the NNAI group and the control group. WMD, weighted mean difference; CI, confidence interval; CNBD, corneal nerve branch density; NNAI, non-neurological autoimmune (diseases).

Finally, twenty-two studies with a total of 1,699 participants (924 in the NNAI group and 775 in the control group) reported on CNBD. The weighted mean difference was −9.89 (95% CI: −14–−5.79, *P* < 0), with significant heterogeneity across studies (I^2^ = 88.4%, Figure 6). Consistently, CNBD (no./mm2) of NNAI patients was significantly lower than that of healthy controls.

### Publication Bias

**Figure 7:**
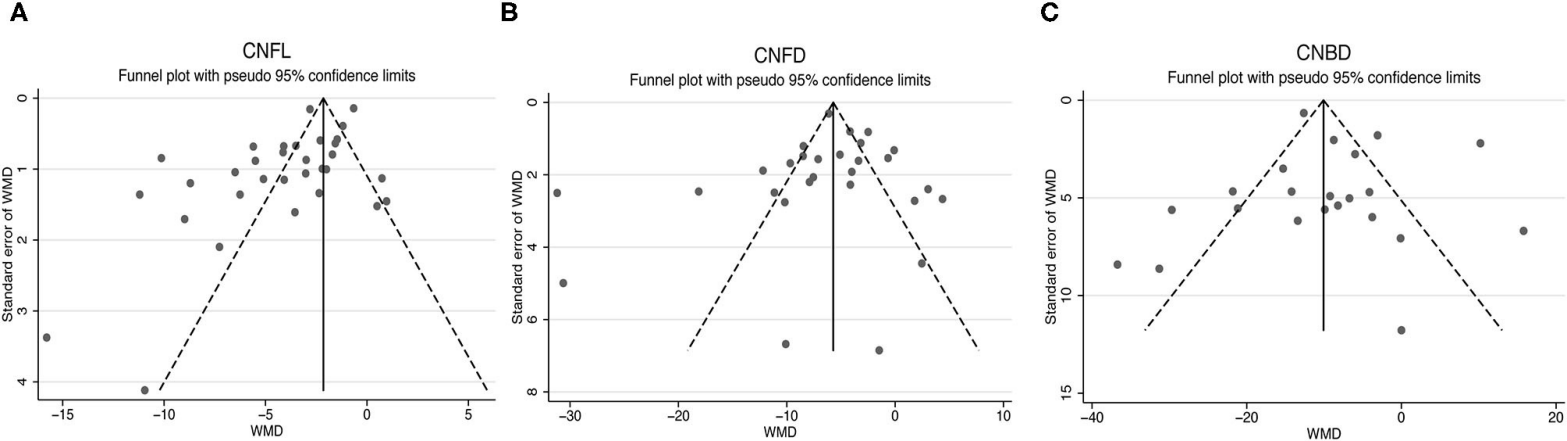
Funnel plots for studies included reported CNFL **(A)**, CNFD **(B)**, and CNBD **(C)**. CNFL, corneal nerve fiber length; CNFD, corneal nerve fiber density; CNBD, corneal nerve branch density; WMD, weighted mean difference.

The publication bias of the studies was shown by funnel plots (Figure 7). The symmetrical funnel plot showed no significant publication bias in the publications reported on CNFD and CNBD. However, the results revealed that studies reported CNFL was mild asymmetry visually, suggesting a publication bias. In addition, Egger linear regression tests and the Begg's rank association tests were performed (Table 2). All other results demonstrated no evidence of significant publication bias except for Egger's test on CNFL. After recalculating the WMD on CNFL using the trim and fill methods, the pooled results were similar to the original results, which means the observed publication bias did not influence the overall results.

**Table 2:** Publication bias measured by Begg's and Egger's tests, WMD (95% CI) recalculated with trim and fill method.

**Subject**	**CNFL**	**CNFD**	**CNBD**
Begg's test	0.195	0.323	0.554
Egger's test	0.001	0.548	0.657
WMD1 (95% CI)^†^	−3.94 (−4.77, −3.12)	–6.62 (−8.40, −4.85)	−9.89 (−14.00, −5.79)
WMD2 (95% CI) ^‡^	−3.81 (−4.64, −2.99)	NA	NA

*CNFL, corneal nerve fiber length; CNFD, corneal nerve fiber density; CNBD, corneal nerve branch; WMD, weighted mean difference; CI, confidence interval; NA, not available*.

† *Original WMD and 95% CI*.

‡ *WMD and 95% CI after using the trim and fill method*.

### Sensitivity Analysis and Subgroup Analysis

**Figure 8:**
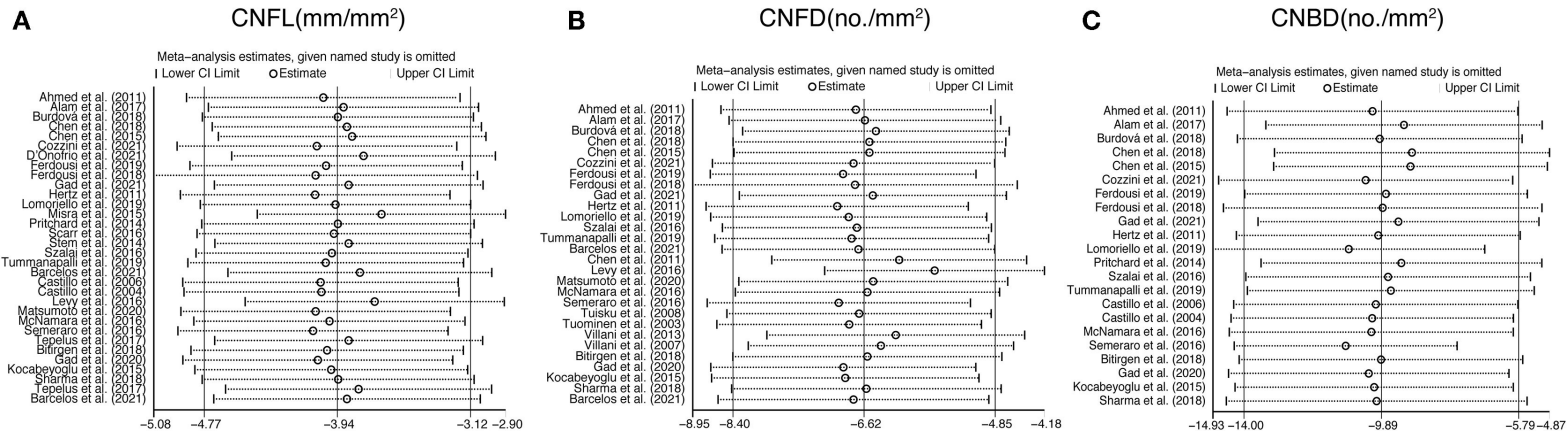
Sensitivity analysis data for studies included reported CNFL **(A)**, CNFD **(B)**, and CNBD **(C)**. CNFL, corneal nerve fiber length; CNFD, corneal nerve fiber density; CNBD, corneal nerve branch density.

To explore the source of heterogeneity, sensitivity analysis was performed. The results revealed that no individual study had an excessive influence on the above-mentioned pooled effect (Figure 8).

Stratifications by age, type of IVCM, software used to measure corneal nerve parameters, and type of NNAI diseases were analyzed due to high heterogeneity. Among studies that reported CNFL, subgroup analysis demonstrated that heterogeneity was reduced for studies grouped by type of IVCM only when using SSCM to record CNFL (I^2^ = 26.1%). Among studies that reported CNFD, subgroup analysis demonstrated that heterogeneity was reduced for studies grouped by the software used only when using built-in software to assess CNFD (I^2^ = 43.1%). And among studies that reported CNBD, heterogeneity was significantly reduced for studies grouped by the software used only when using Image J to assess CNBD (I^2^ = 0%). The detailed results of subgroup analysis are depicted in Table 3.

**Table 3:** Subgroup analysis of CNFL, CNFD, and CNBD by age, type of IVCM, software used, and types of NNAI diseases.

**Subgroup**	**Group by**	**CNFL**			**CNFD**			**CNBD**		
		***N***	**WMD (95%CI)**	***I* ^2^**	***N***	**WMD (95%CI)**	***I* ^2^**	***N***	**WMD (95%CI)**	***I* ^2^**
**Age**										
	10–20	5	−2.32 (−4.06, −0.59)	76.0	5	−3.18 (−6.27, −0.08)	76.3	5	−10.13 (−18.03, −2.24)	73.9
	30–40	8	−3.39 (−4.52, 2.26)	68.9	7	−4.72 (−6.93, −2.52)	74.7	7	−8.14 (−16.10, −0.18)	76.3
	40–50	6	−4.22 (−5.33, −3.12)	80.6	5	−9.38 (−12.59, −6.17)	86.5	5	−18.30 (−24.61, −11.99)	83.9
	50–60	9	−7.01 (−10.47, −3.55)	94.3	9	−8.75 (−15.62, −1.87)	94.6	3	1.61 (−9.44, 12.66)	81.6
	>60	3	−2.85 (−5.26, −0.44)	92.1	/	/	/	/	/	/
**Type of IVCM**										
	LSCM	25	−4.51 (−5.49, −3.52)	94.6	20	−7.44 (−9.50, −5.38)	91.6	15	−13.56 (−17.22, −9.90)	79.3
	SSCM	6	−2.26 (−3.10, −1.43)	26.1	6	−6.25 (−10.84, −1.66)	86.2	6	−2.34 (−9.13, 4.46)	50.9
**Software used**										
	CCMetrics	13	−3.80 (−5.02, −2.58)	80.7	10	−4.81 (−7.75, −1.87)	83.2	11	−15.16 (−21.00, −9.32)	77.2
	Built-in software	/	/	/	2	−8.41 (−16.94, 0.11)	43.1	/	/	/
	ACCMetrics	5	−2.24 (−3.10, −1.39)	73.6	5	−4.98 (−6.53, −3.43)	67.1	5	−10.64 (−16.04, −5.24)	84.8
	Image J	9	−6.19 (−9.04, −3.34)	95.3	5	−7.35 (−16.32, 1.62)	96.9	2	9.87 (5.63, 14.12)	0.0
	Cell Count software	/	/	/	2	−14.95 (−20.75, −9.14)	72.7	/	/	/
**Types of NNAI diseases**										
	Type 1 diabetes	18	−4.14 (−5.14, −3.14)	90.3	13	−4.95 (−6.53, −3.37)	84.1	14	−13.49 (−17.93, −9.05)	83.7
	Sjögren's Syndrome	8	−3.74(−5.71, −1.78)	92.6	10	−11.45 (−18.02, −4.87)	94.1	4	−0.42 (−10.32, 9.48)	82.3

*N, number; CNFL, corneal nerve fiber length; CNFD, corneal nerve fiber density; CNBD, corneal nerve branch density; WMD, weighted mean difference; CI, confidence interval; IVCM, in vivo confocal microscopy; LSCM, laser scanning confocal microscopy; SSCM, slit scanning confocal microscopy; NNAI, non-neurological autoimmune (diseases)*.

### Tortuosity, Reflectivity, and Beadings

**Figure 9:**
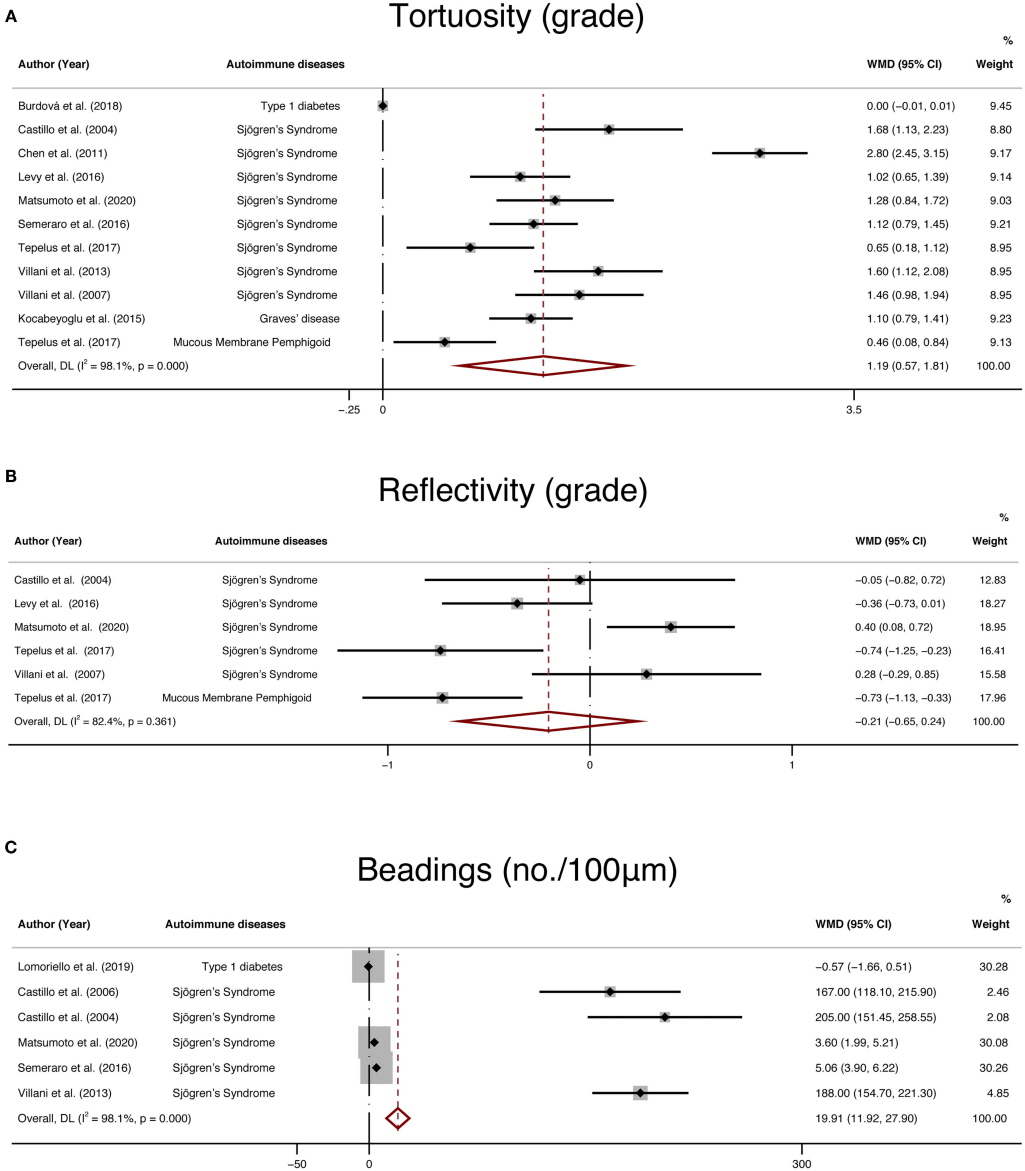
Forest plot of the WMD of tortuosity **(A)**, reflectivity **(B)**, and beadings **(C)** between the NNAI group and the control group. WMD, weighted mean difference; CI, confidence interval; NNAI, non-neurological autoimmune (diseases).

In addition, IVCM enabled en-face examination of corneal nerves. Therefore, tortuosity, reflectivity, and beadings are also widely used to quantify corneal nerve morphology. We collected 11 studies that reported tortuosity, 6 studies that reported reflectivity, 6 studies that reported beadings and performed a meta-analysis. The results demonstrated that the differences in tortuosity (WMD: 1.19, 95% CI:0.57–1.81) and beadings (WMD: 19.91, 95% CI: 11.92–27.9) between the NNAI group and the control group were statistically significant, while the reflectivity (WMD: −0.21, 95% CI: −0.65–0.24) of NNAI patients showed no statistical difference from healthy controls (Figure 9).

**Figure 10:**
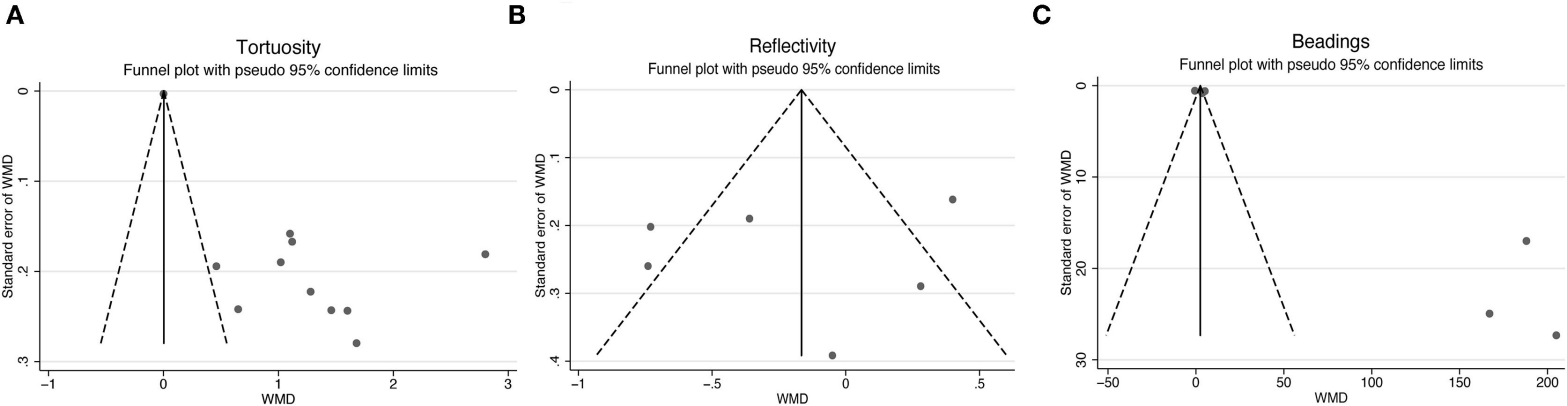
Funnel plots for studies included reported tortuosity **(A)**, reflectivity **(B)**, and beadings **(C)**. WMD, weighted mean difference.

**Figure 11:**
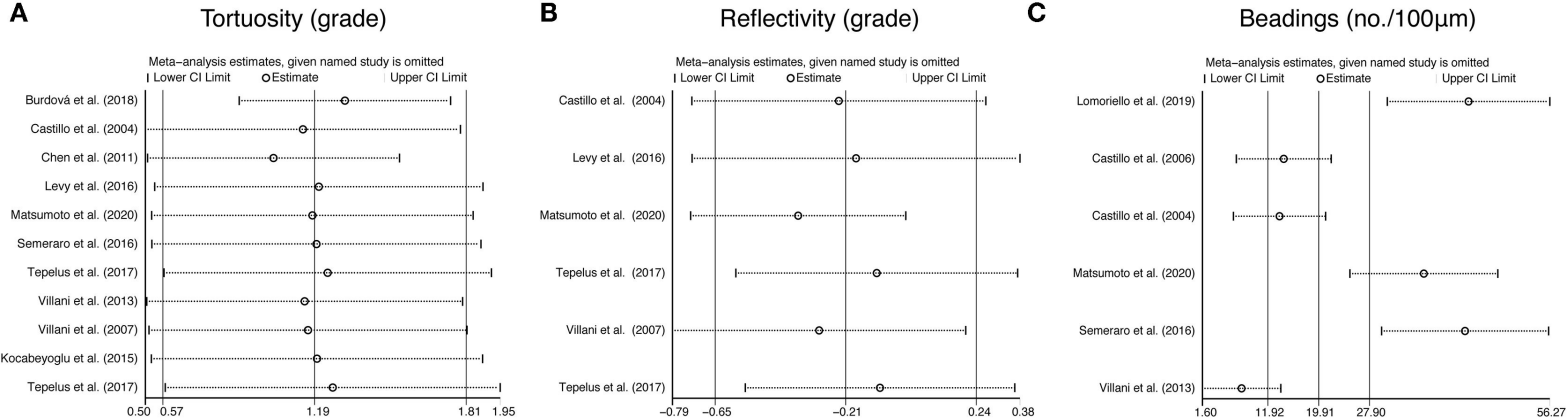
Sensitivity analysis data for studies included reported tortuosity **(A)**, reflectivity **(B)**, and beadings **(C)**.

To further assess the reliability of our results, we also performed funnel plots (Figure 10), as well as Egger's linear regression tests and Begg's rank association tests (Table 4) to estimated publication bias. Sensitivity analysis of studies that reported beadings of corneal nerve per 100 μm showed that four out of six studies may have excessive influence on the above-mentioned pooled effect, and results of tortuosity and reflectivity showed that no individual study had an excessive influence on the above-mentioned pooled effect (Figure 11). Funnel plots of tortuosity and beadings were visually asymmetric, suggesting possible publication bias. Egger's test also showed that there may be a publication bias on studies reported on tortuosity and beadings. After using the trim and fill methods, the pooled result of tortuosity was not changed while that of beadings was quite different from the original results. According to our study, the publication bias did not influence the overall results of tortuosity but did interfere with the overall result of beadings.

**Table 4:** Publication bias measured by Begg's and Egger's test, WMD (95% CI) recalculated with trim and fill method.

**Subject**	**Tortuosity**	**Reflectivity**	**Beadings**
Begg's test	0.815	0.851	0.091
Egger's test	0.000	0.706	0.017
WMD1 (95% CI)^†^	1.19 (0.58, 1.81)	−0.21 (−0.65, 0.24)	19.91 (11.92, 27.90)
WMD2 (95% CI) ^‡^	1.19 (0.58, 1.81)	NA	8.40 (−1.09, 17.88)

*WMD, weighted mean difference; CI, confidence interval*.

† *Original WMD and 95% CI*.

‡ *WMD and 95% CI after using the trim and fill method*.

## Discussion

The cornea, as the front portion of the ocular surface, plays an important role in the visual system. Its integrity is crucial for the health and normal function of the eye, and its delicate mucosal immune system was extremely vulnerable to autoimmune dysregulation so that the cornea is able to detect and repair the damage promptly. It was reported that assessment of corneal nerve parameters has become one of the most common clinical tests to evaluate ocular surface symptoms in many kinds of diseases (63). IVCM provides a direct and non-invasive tool to observe corneal nerve morphology and assess corneal nerve parameters. NNAI diseases, to our knowledge, are a range of diseases with abnormal autoimmune reactions including varied manifestations on the ocular surface. Many researchers reported that the involvement of the cornea may be an initial manifestation of some of the autoimmune diseases and may be sight-threatening if not well treated (64–66). As one of the most densely innervated parts of the human body, the corneal nerve may serve as a marker of some diseases with its morphological alternation.

In the pathology of NNAI diseases, the exact etiological and pathophysiological mechanisms are often unknown. However, many researchers found that elevating inflammatory mediators, such as IL1-beta, IL6, IL8, and TNF-alpha might play an important role in autoimmune patients with small fiber neuropathy. Reducing mechanical nociceptive thresholds and dysesthesias were also found to be associated with higher IL1-beta and TNF-alpha concentrations (67–69). The corneal nerves, as one kind of small nerve fibers, may share the same mechanisms to some extent. Patients with Sjögren's syndrome, systemic lupus erythematosus, or rheumatoid arthritis, for instance, were found to have local increasing lymphocytes in the cornea which implied inflammatory infiltration in corneal nerve fibers (70). Recently, researchers using Mouse models of type 1 diabetes found that decreasing neutrophil infiltration and reducing expression of IL1-beta and TNF-alpha could prevent corneal nerve loss (71, 72). In this way, we speculate that inflammatory mediators may be one reason why a similar pattern of corneal nerve loss occurs in NNAI diseases. Other mechanisms such as metabolic, infectious, and genetic factors may also take part, but the exact pathophysiological mechanisms would need future explorations.

Various works of research had proved that corneal IVCM could be a sensitive evaluation tool in early diabetic peripheral neuropathy and might be clinically useful to diagnosis and surveillance of other neuropathies (48, 73, 74). It is plausible that the alteration of the corneal nerve under IVCM may be a tool to identify NNAI diseases. The other way around, the effect of NNAI diseases on the corneal nerve might be the reason why ocular symptoms were commonly presented among NNAI patients. It is well acknowledged that the corneal nerve helps maintain a well-lubricated and smooth eye surface not only by inducing tear production but also by stimulating the blinking reflex through an elaborate interaction between the corneal surface and lacrimal glands (75). Therefore, damage of the corneal nerve may be associated with the ocular sicca symptoms usually seen and more severe in many NNAI diseases (76–78).

However, many of the previous studies are limited in sample size and their results were contradictory. There is a lack of analytical summary to evaluate the change of corneal nerves in a certain spectrum of NNAI diseases. In this case, a meta-analysis is a powerful tool to summarize results from different studies by providing a more objective evaluation of the major effect with enhanced accuracy and to explain the heterogeneity between different studies. To the best of our knowledge, this is the first meta-analysis to investigate the corneal nerve parameters using IVCM in patients with NNAI and control groups. Our analysis showed significantly decreased CNFL (WMD: −3.94, 95% CI: −4.77–−3.12), CNFD (WMD: −6.62, 95% CI: −8.4–−4.85), CNBD (WMD: −9.89, 95% CI: −14–−5.79) in NNAI groups. However, there was significant heterogeneity of three sets of parameters mentioned above among the studies included. Sensitivity analysis, creation of funnel plots, Egger's test, Begg's test, and the trim and fill methods were performed to confirm the reliability of the results. And the analysis stratified by age, type of IVCM, software used, and types of NNAI diseases, were performed to assess between-study heterogeneity. However, subgroup results showed no potential source of heterogeneity. In the article of Roszkowska et al., it was concluded that corneal nerve changes in diabetes examined by IVCM are related to HbA1c level, diabetes duration, the progress of diabetic retinopathy, and race (79). It is possible that factors such as the severity or duration of NNAI, racial differences in participants, male-female distribution, the acquisition mode with IVCM, or the number of images analyzed per participant, might cause heterogeneity. Due to the incomparability and incompleteness of data, the effect of these above-mentioned potential factors on between-study heterogeneity could not be further examined. All in all, our meta-analysis included thirty-seven studies and with analysis of a large sample size, had shown a significant decrease in CNFL, CNFD, and CNBD among NNAI patients.

In addition, it is interesting that results showed patients with Sjögren's syndrome had a greater reduction in CNFD and minimal impact on CNBD, and consequent comparable reduction in CNFL. In many diseases affecting corneal nerves, CNBD was found to be elevated rather than reduced as subconsciously assumed. For instance, the pattern of corneal nerves appeared to be unique in Parkinson's disease with reduced CNFD and a markedly increased CNBD (80, 81). Similarly, a study demonstrated enhanced CNBD and reduced CNFD and CNFL in patients with painful diabetic neuropathy (82). In addition, increased CNBD was also found to be the first sign to indicate regeneration after simultaneous pancreas and kidney transplantation or continuous subcutaneous insulin infusion in type 1 diabetes (83, 84). All studies mentioned above supported the hypothesis that enhanced CNBD signified some preserved susceptibility of corneal nerve fibers toward regeneration and attempts to repair, but the attempts as yet appeared insufficient to culminate in an increased CNFD or CNFL. Consequently, we consider that CNBD's attempt at regeneration may to some extent compensate for the reduced CNBD by injury. As for CNFL and CNFD, to our knowledge, it is proved that CNFL has been shown to have the best reproducibility and consistency compared to CNFD and CNBD for detecting early preclinical small fiber damage (54, 79, 85, 86). This may indicate that CNFL is most susceptible to damage from various diseases, but our results presented contradictory. It is strange and hard to explain, and maybe knowing the exact pathology of Sjögren's syndrome would help explain it. However, at present, studies on corneal nerve alternation of patients are mainly *in vivo* studies, which means that we can basically only carry out some non-invasive detections like corneal confocal, corneal sensation, biological fluid detection, etc. Although these detections prove to be very promising ways to an early small fiber neuropathy diagnosis (87), the exact pathophysiology and signaling pathways activated in diseases remain unknown due to invasive procedures that cannot be performed *in vivo*. Moving forward, more future research is needed for a deeper understanding.

Besides CNFL, CNFD, and CNBD, tortuosity, reflectivity, and beadings are also important parameters to describe corneal nerve morphology. According to our analysis, the corneal nerve of the NNAI group presented more beadings per 100 μm (WMD: 19.91, 95% CI: 11.92–27.9) and was more tortuous (WMD: 1.19, 95% CI:0.57–1.81) than that of the control group, while there seemed to be no statistical difference on corneal nerve reflectivity (WMD: −0.21, 95% CI: −0.65–0.24, *P* = 0.361) between two groups. However, the results of subjective parameters like tortuosity and beadings in our analysis seem to be less convincing according to publication bias analysis. There could be due to many reasons. One of the reasons may be that these subjective parameters are infrequently reported in the included literature, resulting in a small sample size of data. Another reason may be that measurement of subjective parameters is not uniform across studies. For instance, some studies reported corneal nerve tortuosity according to previously validated grading scales, while others used tortuosity coefficient (47). Besides, the interpretations of the results by these subjective parameters rely a lot on researchers' subjective judgment and observers' experience, which made the results less comparable.

Nevertheless, we can't deny the promising function of subjective parameters in predicting corneal nerve neuropathy. Indeed, according to research examining corneal nerves in patients with type 2 diabetes (88), the size and number of beadings had the best sensitivity and specificity to predict the dysfunctions of the peripheral neuropathy compared with CNFD, CNFL. Similarly, a previous study among glaucoma patients showed that tortuosity and beadings directly correlated with corneal nerve function (89). In recent years, software and methods have been developed to obtain more objective and reproducible evaluations of tortuosity (90, 91). For example, a study proposed an automatic algorithm that was able to correctly trace more than 80% of the recognizable nerve fibers in the images and proved its clinical validity regarding tortuosity measure (92). We believe that in the future, more accurate software will help make these subjective parameters more comparable among various studies and more practical in clinical performance.

The present study has some limitations that should be considered. Firstly, the types of NNAI included in our study were mostly typed 1 diabetes and Sjögren's Syndrome, which might not be representative of NNAI in general. Many other NNAI diseases were reported presenting ocular manifestation as the initial manifestation like rheumatoid arthritis and systemic lupus erythematosus. It is reasonable to infer that alteration in corneal innervation also occurs in these diseases, but it is a pity that we did not find qualified studies for every NNAI disease that could be included in the meta-analysis. And we look forward to more research about morphological alternation of the corneal nerve of NNAI so we may draw a more reliable conclusion. Secondly, although IVCM has already been widely used in clinical practice, there is still a lack of a gold standard for corneal nerve parameters. For example, the majority of studies have defined CNFL as the total length of nerves visible within a defined area in mm/mm^2^ while some only measured nerve branches longer than 50 μm or analyzed the total length of nerves within a frame (93–97). Other factors contributing to the non-uniform assessment may include. (1) Each image captured by IVCM represents only approximately 0.2% of the average corneal surface which might give out non-representative images and result in misleading inferences (7, 98). (2) A possible correlation between myopic refractive error and CNFL might be neglected among our included articles that assess corneal nerves (99). (3) According to instrument design, IVCM can be generally divided into tandem scanning confocal microscopy, laser scanning confocal microscopy, and slit scanning confocal microscopy (100). Different kinds of confocal microscopy are equipped with different field brightness and contrast which may affect the apparent thickness of corneal nerves, particularly when they approach the limit of resolution, thus influencing the uniformity among different studies (97, 101). (4) IVCM image processing could be performed by different methods, including manual tracing, ImageJ, the CCMetrics system, the ACCMetrics system, etc (102, 103). The inconsistency of image-processing methods and the subjectivity during the image-analyzing procedure among different studies may also result in significant discrepancy and heterogeneity.

In conclusion, this meta-analysis suggested that corneal nerve parameters (CNFL, CNFD, CNBD) might be clinical markers for NNAI diseases, while our analysis of other morphology indicators (tortuosity, reflectivity, beadings) lack reliable conclusion from the included studies. Future longitudinal studies could delve into the role of IVCM as a promising way to diagnose and evaluate NNAI diseases.

## Data Availability Statement

The original contributions presented in the study are included in the article/supplementary material, further inquiries can be directed to the corresponding authors.

## Author Contributions

YG, XL, and XY conceived of the study, carried out the literature search, extracted the data, and performed the statistical analysis. NY and WK conducted the quality assessment. YG, XL, and QQ were involved in revising and modification of the manuscript. KW and MC directed the project, reviewed, and revised the manuscript. All authors have contributed significantly and agree with the content of the manuscript. All authors read and approved the final manuscript.

## Funding

This study was supported by the Zhejiang Provincial Natural Science Foundation of China (Nos. LGF20H120003 and LY19H120006) and the National Natural Science Foundation of China (No. 82171045).

## Conflict of Interest

The authors declare that the research was conducted in the absence of any commercial or financial relationships that could be construed as a potential conflict of interest.

## Publisher's Note

All claims expressed in this article are solely those of the authors and do not necessarily represent those of their affiliated organizations, or those of the publisher, the editors and the reviewers. Any product that may be evaluated in this article, or claim that may be made by its manufacturer, is not guaranteed or endorsed by the publisher.
